# A rare case of plexiform schwannoma on the foot

**DOI:** 10.1002/ccr3.4234

**Published:** 2021-05-25

**Authors:** Farnaz Araghi, Mohammadreza Tabary, Kambiz Kamyab, Mohammad‐Mehdi Forouzanfar, Reza M. Robati

**Affiliations:** ^1^ Skin Research Center Shahid Beheshti University of Medical Sciences Tehran Iran; ^2^ School of Medicine Tehran University of Medical Sciences Tehran Iran; ^3^ Department of Dermatopathology Razi Hospital Tehran University of Medical Sciences Tehran Iran; ^4^ Department of Emergency Medicine Shohada Tajrish Hospital Shahid Beheshti University of Medical Sciences Tehran Iran; ^5^ Department of Dermatology Loghman Hakim Hospital Shahid Beheshti University of Medical Sciences Tehran Iran

## Abstract

Plexiform schwannoma is an uncommon soft tissue tumor that could even rarely presented on the foot and toes.

## Graphical abstract

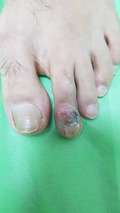

## 1 INTRODUCTION

Plexiform schwannoma mainly presents as an asymptomatic nodule located on the head, neck, or upper extremities. The presentation of this tumor on the dorsum of the foot is very rare. Herein, we describe a rare case of pretty large plexiform schwannoma on the dorsal surface of the toe.

Plexiform schwannoma is considered an uncommon benign peripheral nerve sheath tumor that stems from the slow proliferation of Schwann cells in a meshwork configuration.^1^ This tumor has been observed in men and women equally and mostly presents during childhood. The size of the tumor is less than 2 cm in most cases. Possible risk factors such as positive family history, trauma history, or neurofibromatosis type 2 may be observed in cases.^2^ Plexiform schwannoma is mainly presented as a slow‐growing asymptomatic solitary nodule located on the head and neck region or upper extremities.^3^ Rarely, plexiform schwannoma can present on the lower extremities particularly on the foot. According to previous reports, the presentation of this tumor on the dorsum or interdigital space of the foot seems to be the rarest manifestation.^4^ Herein, we describe a rare case of pretty large plexiform schwannoma on the toe in a male patient.

## 2 CASE PRESENTATION

A 33‐year‐old male patient was referred to our dermatology clinic due to the growing soft tissue mass on his foot. Initially, the mass appeared on his second toe of the right foot 20 years ago. Recently, he has noted a progressive growth in the bulk for the last 6 months. He denied any significant past medical history or drug history. He did not mention any relevant familial history, as well.

**FIGURE 1:**
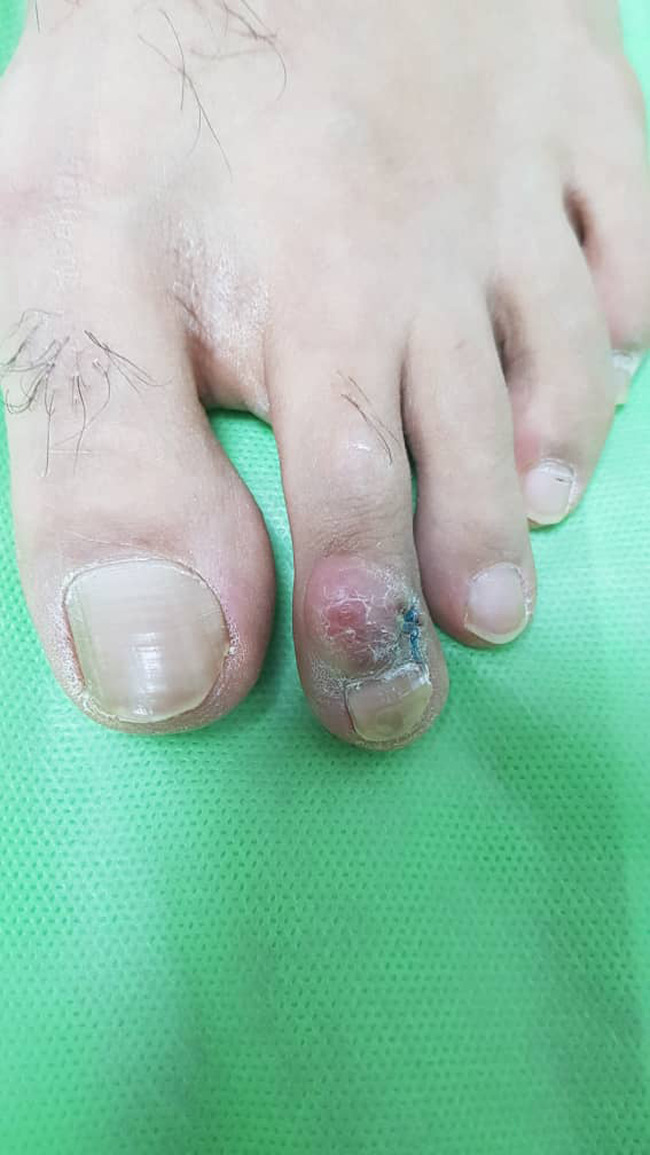
A nontender erythematous nodule on the phalanx of the second right toe

In the physical examination, there was a nontender erythematous nodule on the phalanx of his second right toe. The nodule was immobile and measured 3 cm in diameter (Figure 1). No similar lesions were observed on any other parts of his body. Accordingly, X‐ray imaging confirmed that the nodule was arising with the soft tissue and no bone involvement was detected.

**FIGURE 2:**
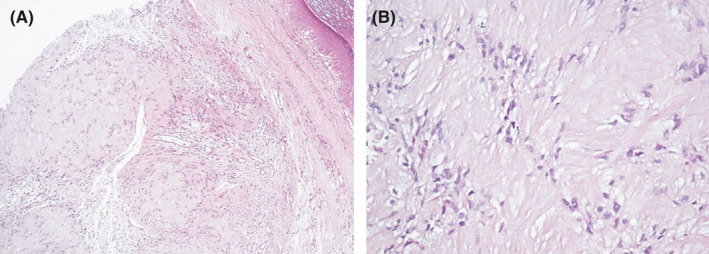
A, B, The areas of nuclear palisading forming verocay body appearance of intervening stroma between the nodules composed of a loose meshwork of spindle cells with microcystic change and scattered inflammatory cells

**FIGURE 3:**
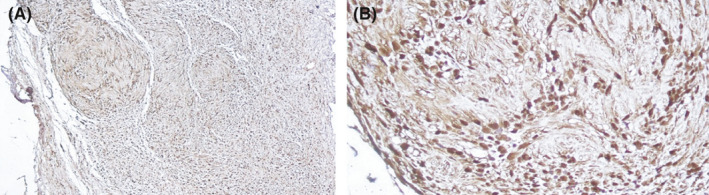
A, B, The immunostaining reported positive was for S‐100

Punch biopsy was taken from the nodule while considering myxoid cyst, neuromas, and giant cell tumor of tendon sheath as the differential diagnoses. Microscopic examination revealed a portion of plexiform neoplasm in the dermis composed of multiple nodules of proliferated spindle cells with wavy nuclei in a fibrillary matrix. There were areas of nuclear palisading forming verocay body appearance of intervening stroma between the nodules composed of a loose meshwork of spindle cells with microcystic change and scattered inflammatory cells (Figure 2). In addition, immunostaining was performed for S‐100 and Epithelial Membrane Antigen (EMA) and reported positive and negative, respectively (Figure 3). These histopathology features were in favor of plexiform schwannoma. Finally, the patient was referred to an orthopedic surgeon in order to excise the lesion precisely.

## 3 DISCUSSION

Herein, we reported a case of plexiform schwannoma with an unusual size that developing in an unusual location. Generally, Schwann cell tumors are categorized as neurofibromas, schwannomas, and malignant peripheral nerve sheath tumors (MPNSTs). In addition, these tumors can be considered as a manifestation of genetic diseases, such as Neurofibromatosis type 2 (NF‐2), carney complex type 1, and schwannomatosis. Moreover, these tumors have been rarely reported in unusual locations such as gingiva.^5^, ^6^

Schwannoma is considered as a benign peripheral nerve sheath tumor that stems from the slow proliferation of Schwann cells. Schwannoma mostly appears as a solitary nodule with slow growth which can be histologically classified into cellular, plexiform, and melanotic variants.^2^

The plexiform variant is a benign tumor, which develops in a plexiform pattern; nevertheless, the malignant transformation is rarely reported.^5^ Clinically, this tumor often develops as a solitary subcutaneous or intradermal nodule and grows in a small size, the greatest diameter of which is mostly lower than 2 cm.^7^ Imaging particularly, MRI can be helpful in diagnosis and localization of the lesion before the treatment; however, the diagnosis cannot be confirmed without histopathological studies.^8^

From the histopathological view, various nodules which locate intradermally or subcutaneously consist of cellular Antoni A, in which areas with both nuclear palisading and verocay bodies are detected. In contrast to schwannomas, plexiform schwannomas develop in a plexiform configuration including various interconnecting nodules and fascicles in Antoni A areas.^9^ Plexiform neurofibroma could be considered as a differential diagnosis of plexiform schwannoma from a histological aspect. However, plexiform schwannomas are made of Schwann cells which can be detected via S100 protein staining.^10^, ^11^

## 4 CONCLUSION

Plexiform schwannoma is a rare benign tumor that can rarely occur on the toes. It is recommended to keep in mind this diagnosis facing bizarre large soft tissue tumors on the foot or toes. Histopathological studies are required to avoid misdiagnosis with other lesions. The treatment is limited to surgical treatments and complete excision is recommended in order to prevent the recurrence.

## CONFLICT OF INTEREST

None declared.

## AUTHOR CONTRIBUTIONS

FA: Acquired the data and prepared the manuscript. MT: Acquired the data and wrote the manuscript. KK: performed the laboratory test and wrote the manuscript. M‐MF: acquired the data and wrote the manuscript. RMR: served as the corresponding author and designed and supervised all the aspects and contributed to manuscript editing. All authors: contributed sufficiently and met the criteria for authorship.

## ETHICAL APPROVAL

The ethical issues were completely considered to prepare this case report according to our institution's ethical board guidelines. Moreover, this article was prepared regarding the declaration of Helsinki.

## CONSENT STATEMENT

Published with written consent of the patient.

## Data Availability

The data are also available on request.
